# Influence of Geographic Location in Modeling Blood Pesticide Levels in a Community Surrounding a U.S. Environmental Protection Agency Superfund Site

**DOI:** 10.1289/ehp.8154

**Published:** 2005-08-17

**Authors:** Shannon H. Gaffney, Frank C. Curriero, Paul T. Strickland, Gregory E. Glass, Kathy J. Helzlsouer, Patrick N. Breysse

**Affiliations:** 1 Department of Environmental Health Sciences, Johns Hopkins Bloomberg School of Public Health, Baltimore, Maryland, USA; 2 ChemRisk, Inc., San Francisco, California, USA; 3 Department of Biostatistics,; 4 Department of Molecular Microbiology and Immunology, and; 5 Department of Epidemiology, Johns Hopkins Bloomberg School of Public Health, Baltimore, Maryland, USA

**Keywords:** biomarkers, DDE, DDT, dieldrin, geostatistics, organochlorines, spatial statistics, Superfund

## Abstract

In this study we evaluated residential location as a potential determinant for exposure to organochlorine compounds. We investigated the geographic distribution characteristics of organochlorine levels in approximately 1,374 blood samples collected in 1974 from residents of a community with a potential organochlorine source. Street addresses of Washington County, Maryland, residents were obtained and geocoded in a geographic information system. We used multivariate linear regression models to characterize the blood organochlorine levels of these residents that had been analyzed as part of previous studies using both environmental- and individual-level covariates. This was done to evaluate if the geographic distribution of blood levels in participants was related to the environmental source in the community. Model inference was based on generalized least squares to account for residual spatial variation. A significant inverse relationship was found between blood dieldrin levels and residential distance from the potential source. For every mile of distance from the source, blood dieldrin levels decreased 1.6 ng/g in study participants (*p*-value = 0.042), adjusting for age, sex, education level, smoking status, and drinking water source. 1,1-Dichloro-2,2-bis(*p*-chlorophenyl)ethylene (DDE) levels in the blood did not change significantly based on residential distance from the source, taking the same covariates into account. However, these results are limited by the inability to account for several potential confounders. This study demonstrates that spatially distributed covariates may play an important role in individual exposure patterns. Spatial information may enable researchers to detect a potential exposure pattern that may not be revealed with only nonspatial variables.

Spatial information has long been used to study the environmental contamination patterns of persistent organochlorine pollutants. These environmental data are often used as surrogates for exposure experienced by the studied community. However, organochlorine levels may also be measured in serum, providing a more accurate account of exposure. Because we can also link spatial information, such as location of residence, to blood donors, spatially evaluating biomarkers of exposure is a logical extension to investigating spatial patterns in environmental media.

In the early 1930s, a large chemical company built a 19-acre facility in the city of Hagerstown in Washington County, Maryland, for the production of fertilizers and formulation of pesticides, including 1,1,1-trichloro-2,2-bis(*p*-chlorophenyl)ethane (DDT) and dieldrin. Site investigations since the 1970s have indicated the presence and migration of organochlorine pesticides, such as DDT and dieldrin, as well as other toxicants to off-site areas, and the U.S. Environmental Protection Agency (EPA) placed the site on the National Priority List for cleanup in 1997 as a Superfund site [Maryland Department of the Environment (MDE) 1993, 1994, 1995, 1996; Roy F. Weston Inc. 1997]. A more detailed description of the site can be found elsewhere (Henshaw 2004; Henshaw et al. 2004).

In this study we investigated the relationship between the location of the homes of Washington County residents, their proximity to the Superfund site, and the levels of organochlorine compounds in the blood of the residents. Spatial and other known covariates are evaluated in multivariate linear regression models of blood organochlorine levels. Residual spatial variation from these regressions that is not accounted for by the model is further evaluated using generalized least squares (GLS) with a spatial correlation structure so as to provide proper estimation of effect standard errors and corresponding tests of significance.

## Materials and Methods

### Data.

More than 20,000 adult Washington County residents (roughly one-third of the county population) signed written consent forms to donate blood for research purposes as part of the Campaign Against Cancer and Stroke (CLUE I) in the fall of 1974 (Comstock et al. 1991). A subset of these samples (*n* = 1,391) was analyzed for organochlorine compounds to examine the association between concentrations of these compounds and subsequent cancer (Cantor et al. 2003; Helzlsouer et al. 1999; Rothman et al. 1997). All samples were assayed for DDT, 1,1-dichloro-2,2-bis(*p*-chlorophenyl)ethylene (DDE), and polychlorinated biphenyls (PCBs). About half of these samples were also assayed for additional organochlorines such as dieldrin. Details concerning the blood collection, storage, and analytical methods have been published elsewhere (Cantor et al. 2003; Helzlsouer et al. 1999; Rothman et al. 1997). This study was approved by the Johns Hopkins Committee on Human Research.

We used 1,391 blood samples assayed for organochlorine concentrations. Blood-sample organochlorine concentrations from four subjects whose blood was assayed twice were averaged for analysis purposes. Thirteen subjects were found to reside outside of Washington County at the time of blood draw and were removed from the sample pool. In addition, one sample had reported DDE values > 2.25 times higher than the next highest reported value, and was therefore considered a reporting error and removed from analysis. Ultimately, a total of 1,374 samples were considered valid for this study.

Street addresses and ZIP codes of the study participants were collected as part of the CLUE campaign. ArcGIS 8.2 (Environmental Systems Research Institute, Inc. 2002) software was used to geocode the addresses, providing a corresponding set of longitude and latitude coordinates. The geocoding process employed several base maps (Boscoe et al. 2002; McElroy et al. 2003). StreetMap 2000 (Environmental Systems Research Institute 2000), the U.S. Census Bureau 2000 Topologically Integrated Geographic Encoding and Referencing (TIGER) street maps (U.S. Census Bureau 2002), and the Delorme Street Atlas software (Delorme 2003) were all used to maximize geocoding results. In situations where several study participants had the same residential address, such as in an apartment building, the geocoded coordinates were altered slightly by adding 1-foot increments to each of the longitude and latitude coordinates, ensuring that all participants had a unique location and that no two participants were grouped as one when mapping. The Superfund site address was also geocoded. Details regarding this geocoding process are reported elsewhere (Henshaw 2004).

In addition to geocoded address information, we obtained information on participant demographics, such as age, race, sex, education level, marital status, and district average socioeconomic status; variables that have been shown to be predictive of blood organochlorine levels, such as smoking status (current smoker at the time of blood draw); and drinking-water source (municipal/well/spring) (Acquavella et al. 1986; Fitzgerald et al. 1999; Glynn et al. 2003; James et al. 2002; Moysich et al. 2002; Rivero-Rodriguez et al. 1997; Soliman et al. 2003; Sweeney et al. 2001; Wariishi et al. 1986). These data were abstracted from CLUE-based questionnaires and a private Washington County census. The spatial variables of direction and distance from the Superfund site to the residence as well as urban/rural location of residence were created and added to the list of potential covariates. The distance-to-site variable was created to represent a proxy for unknown and unmeasured unique contributions from the source. Urban residence is defined in this study as living within 1.5 miles of the center of Hagerstown. A direction variable was created as a series of indicator variables denoting 24 directional bins (every 15 degrees) around the site. This direction variable was then considered a potential effect as an interaction term, with distance thus allowing a separate distance effect for each direction.

Because DDT breaks down to DDE in the blood, DDE levels were chosen to represent DDT exposure [Centers for Disease Control and Prevention(CDC) 2003]. Total PCBs used for analysis in this study refers to the sum of PCB congeners 105, 118, 146, 153, 156, 170, and 180. Dieldrin was also chosen for analysis to represent an additional compound that was used at the Superfund site.

Blood samples were nonfasting. All compounds were analyzed both unadjusted for lipid content and lipid-adjusted using the method of Phillips et al. (1989). The analytical labs reported levels below the official limits of detection (LODs); reported levels were used because they are considered more valid than an interpolated value (Cantor et al. 2003; Rothman et al. 1997). The LOD divided by the square root of 2 was used for the unreported values below detection (< 5% of the samples were below the LOD) (Hornung and Reed 1990).

### Statistical analysis.

Levels of DDE, PCBs, and dieldrin in participants’ blood were mapped using their geocoded coordinates to study the spatial distribution of the levels of these organochlorines and their possible relationship to the Superfund site. Spatial structure in the levels of organochlorines was further explored using estimated semivariograms (Cressie 1991). Semivariograms were also estimated for regression residuals as a diagnostic check on the independence assumption inherent in ordinary least squares (OLS) inference.

Multivariate linear regression was used to develop models that best describe the blood levels of each organochlorine, both lipid adjusted and unadjusted. These models are of the form


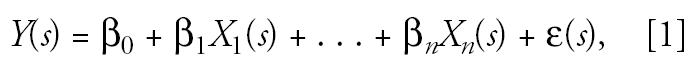


where *s* denotes spatial coordinates, *Y*(*s*) represents blood organochlorine levels of participants residing at location *s*, *X*_1_(*s*) . . . *X_n_*(*s*) are covariates (including possible interactions) indexed by location *s*, β_1_ . . . β*_n_* values are their associated effects, and β_0_ is the baseline intercept. The residual error term ɛ(*s*) was assumed to be normally distributed with a zero mean and constant variance. To further account for possible residual spatial variation, residuals were allowed to be spatially dependent by parameterizing their correlation as a decreasing function of the distance between their locations. In the geostatistical literature, model 1, with these specifications, is known as a universal kriging model commonly used for spatial prediction at unobserved or unmeasured locations (Cressie 1991).

We began to select models for blood levels of each organochlorine by running all possible models derived from each combination of covariates considered as regression main effects as well as investigating univariate relationships between each covariate and the outcome variable. All covariates were checked for colinearity, and those found to be correlated with one another were evaluated separately in the models to determine which were the best predictors. The fraction of variance explained by the model adjusted for the number of explanatory variables (adjusted *R*^2^) was used to rank model performance. The top-performing portion of models was then investigated further for significant interactions among the included covariates. The final models were chosen based on model parsimony and scientifically meaningful interpretations. All exposure determinants, geographic or not, were considered on an equal setting before developing the regression models.

All regression inference at this step was based on OLS regression assuming uncorrelated or independent residuals, which is possibly not a valid assumption. If residual spatial variation exists (an assumption that can be evaluated), then OLS estimates and corresponding tests of significance can lead to invalid results (Diggle et al. 1998a). We used OLS because the objective was first to arrive at a manageable set of plausible final models and then to investigate and correct for residual dependence. OLS methods for estimating regression parameters in models with dependent residuals could lead to spurious significant inclusion of covariates, which would then be reevaluated adjusting for residual spatial dependence (Diggle 2000; Diggle et al. 1998a).

The final models were adjusted for possible residual spatial variation (Carroll et al. 1988; Cressie 1991). Semivariograms were estimated from the OLS model residuals as a diagnostic check for residual spatial variation (Cressie 1991). This was affirmed in all models considered, and we selected the exponential spatial correlation function, routinely applied in spatial statistics, as the function best characterizing the residual spatial variation (Cressie 1991). We also examined directional dependence in the residual spatial variation (anisotropy) by estimating directional dependent semivariograms. The results consistent across all models suggested that the assumption of spatial isotropy better characterized the residual variation. Fitting individual variograms to each contaminant also allows for differences in spatial dispersion of the contaminant that could result from differing chemical properties and uses in production.

We then jointly reestimated the parameters of interest quantifying the covariate effects, β_0_, . . . β*_n_*, with the exponential spatial correlation parameters (range, sill, and nugget in geostatistical terminology) using maximum likelihood, yielding GLS estimates for covariate effects (Diggle et al. 1998b). The GLS estimated standard errors were then used to update tests of significance. We also analyzed transformed blood organochlorine outcomes using the Box-Cox family of transformations, *g*(*Y*) = (*Y*^γ^ − 1)/γ, with *Y* representing an organochlorine compound and γ a parameter of the likelihood to be estimated (Christensen et al. 2001). Two possible transformations to note are γ = 1, no transformation, and γ = 0, natural log transformation. Throughout the analyses, the maximum likelihood estimate for γ was consistently close to one. All regressions were therefore analyzed on the original scale.

All statistical analyses were performed using the R statistical computing environment with the contributed package, geoR, for geostatistical operations (R Development Core Team 2003; Ribeiro and Diggle 2001).

## Results

Demographic information regarding the study population is given in Table 1. The mean age of the participants was 53 years. The participants were 58% male, 19% were current smokers, 98% were white (2% African American), and the mean education level was between 11 and 12 years. Because there were so few African Americans, the results are limited to only the white population (*n* = 1,350).

Table 2 summarizes the blood DDE, total PCB, and dieldrin levels in the participants of the study. The mean levels of DDE, total PCBs, and dieldrin in the blood of Washington County residents adjusted for lipid content were 3023.5, 771.5, and 113.07 ng/g, respectively.

Approximately 96% of the addresses were geocoded successfully. Aside from clustering of residences in accordance with population density, spatial patterns were not apparent. The 50 addresses that were not geocoded, and hence removed from the analysis, consisted mainly of rural routes and post office boxes that the base map was unable to locate (Hurley et al. 2003; McElroy et al. 2003). They showed no distinct patterns with respect to covariates such as age, education, sex, smoking status, or blood organochlorine levels and were from various ZIP codes, uniformly dispersed across the county. Their exclusion from the analysis, therefore, is not expected to introduce bias.

From the exhaustive search of all covariates, we chose plausible regression models for each organochlorine based on their ability to predict model variability. Spatial dependence was found in the residuals of all organochlorines in this step, as diagnosed by their estimated residual semivariograms. Parameter estimates and tests of significance were adjusted for this residual spatial dependence using the GLS-based approach outlined in “Materials and Methods.”

Table 3 gives the results of the adjusted (multivariate) models used to describe the blood levels of DDE, total PCBs, and dieldrin in the Washington County study population. The impact each covariate had on the blood organochlorine level alone was also measured using univariate GLS regression (results listed in Henshaw 2004). Although all covariates mentioned in “Materials and Methods” were evaluated in the regression analysis, only those covariates that were part of the most predictive model of blood organochlorines are presented in Table 3.

Age, sex, smoking status, education, drinking water source, and distance to the Superfund site improved the overall fit of the model of blood DDE levels. Women, non-smokers, and city water drinkers had statistically significantly less DDE in their blood than did men, smokers, and those who drink spring or well water, respectively, when all other covariates were controlled. DDE levels also increased significantly with age. No statistically significant association was found between the level of DDE in the blood and distance of the residence from the Superfund site.

After adjusting for age, sex, smoking status, education, and drinking water source, a statistically significant negative association was found between dieldrin levels in blood and the residential distance from the Superfund site. The only significant predictors of blood dieldrin levels were smoking status and drinking spring water versus city water. Furthermore, smokers tended to have significantly less dieldrin in their blood than did nonsmokers. Nonetheless, the results of this dieldrin model suggest that those who lived closer to the site had higher levels of dieldrin in their blood than did those who lived farther away. If the trend were assumed to be linear, there would be a 1.6 ng/g decrease in blood dieldrin levels for every mile a residence was located away from the site. Follow-up analysis using half-mile increments for distance suggests that linearity in the effect of distance to the site is supported more at distances near the site. However, the linear relationship appears to be weak because it held true only within the first half-mile increment. When the distance variable was broken into mile increments, a linear relationship was not seen.

No relationships between distance to the Superfund site and blood levels of total PCBs were found (Table 3). The spatial covariate, urban versus rural residence, was marginally predictive of lipid-adjusted total PCB levels in blood when adjusting for age, sex, education, smoking status, and drinking water source (*p* < 0.1). Blood levels of total PCBs in participants living within 1.5 miles of the center of Hagerstown were lower than in those living outside of Hagerstown, holding age, sex, education, smoking status, and drinking water source constant. The association was not significant for lipid-unadjusted blood total PCB levels. In addition, while adjusting for other explanatory variables, men, smokers, and well-water drinkers had marginally higher blood PCB levels than did women, nonsmokers, and those who drink city water, respectively. However, only the association with sex was statistically significant. Finally, a positive association with age and a negative association with years of education and blood PCBs were found, although neither of these relationships is statistically significant.

After correcting for spatially dependent residuals, most model parameter estimates were not changed significantly. However, those covariates that bordered on statistical significance (i.e., *p*-values ~ 0.05) were sensitive to correcting for spatially dependent residuals. For example, a statistically significant urban/rural residence relationship with lipid-adjusted total PCBs was found in OLS regression but became statistically insignificant after correcting for spatial dependence in the residuals.

## Discussion

In this study we investigated the importance of evaluating spatial covariates and taking into account residual spatial dependence in regression models attempting to explain levels of contaminants in humans. Spatial information is more commonly used in evaluating environmental contamination but is often overlooked in studies modeling the same contaminants in humans, despite the fact that biomarkers are indicators of exposure. Results of this study indicate that models for blood organochlorine levels can benefit by including spatial information.

Results suggest that residential location may be a potential exposure determinant of organochlorine levels in human blood as biomarkers of exposure to persistent organochlorine compounds in Washington County, Maryland. A significant association is present between blood dieldrin levels and residential distance from the Superfund site. However, an association between residential location and the Superfund site in the county was not found with blood DDE levels. In fact, DDE levels in blood increased with distance from the site instead of decreasing, as anticipated. One possible reason for this pattern may be that DDE is a widespread compound that can be found in the blood of > 90% of the U.S. population, whereas dieldrin was not as commonly found in the environment and in human blood (CDC 2003; Longnecker et al. 1997). In addition, DDT was most likely used often and all over the county before the 1970s, in agricultural occupations. Therefore, there may have been multiple nonpoint sources of exposure to DDT in the study population. It is important to note that the mean levels of DDE in this population are > 10 times the Second National Health and Nutrition Examination Survey (NHANES II) reported national background level of 297 ng/g in 1999–2000 (CDC 2003). The results presented in this article suggest that this widespread use of DDT may be a much larger contributor to internal dose than any increase in body burden associated with living close to the Superfund site. Dieldrin, on the other hand, had more limited use for termite control. Therefore, the site may have been the primary source of dieldrin exposure, resulting in higher blood levels for those individuals living closer to the site.

Further research is needed to determine the validity of the association between blood dieldrin levels and the Superfund site. Not only is the statistical significance of this association marginal, but also the model is based on a sample less than half the size of that for DDE. Furthermore, the model found smoking to be negatively associated with blood dieldrin levels. No other studies in the literature have suggested such an association with smoking, and therefore more research into this finding is warranted. Overall, the results are inconclusive as to whether there is a direct relationship between residential distance to the Superfund site and levels of organochlorines in the blood of the participants.

For the most part, the covariates found to be associated with blood organochlorines in this study are consistent with the literature. For instance, other studies have found that blood organochlorine concentrations were positively associated with age or current smoking status (Fitzgerald et al. 1999; Glynn et al. 2003; Pereg et al. 2002; Sala et al. 1999). Glynn et al. (2003) and Sala et al. (1999) also report that place of residence influences blood levels of organochlorines. The positive age association can be explained by the fact that older people have had a longer duration of exposure, which is reflected in their body burden of organochlorines. Smokers may have higher exposure to blood organochlorines due to the constant hand-to-mouth activity. It also makes sense that those people who rely on wells or springs for drinking water have higher levels of organochlorines in their blood, because the site has been shown to have polluted the surrounding waterways, and the geology of the area is such that surface water can easily contaminate the groundwater (MDE 1993, 1994, 1995, 1996).

The literature regarding certain covariates evaluated in this study is somewhat inconsistent. For instance, we found that men have higher levels of blood organochlorines than women do, perhaps because lactation may lower the organochlorine body burden in women. Some studies have reported similar findings (Stehr-Green 1989; Wolff and Anderson 1999), whereas others observed that women had higher levels than men or that there was no association with sex (Bertram et al. 1986; Sala et al. 1999; Wariishi et al. 1986). A potential reason for this inconsistency could be that sex does not always account for differences in occupation and body mass index (BMI), because these are both potential confounders in this relationship.

An additional limitation is that information on possible confounders or effect modifiers is not complete. For example, the CLUE questionnaire did not obtain height and weight measurements, and therefore BMI could not be considered even though it is a predictor of blood organochlorine levels (Glynn et al. 2003; James et al. 2002; Pelletier et al. 2002; Sala et al. 1999; Schildkraut et al. 1999). Other variables such as breast-feeding history, weight loss, and occupational and home exposure to pesticides have also been significant predictors of blood organochlorine levels (Glynn et al. 2003; Hernandez-Valero et al. 2001; Sala et al. 1999; Soliman et al. 2003; Wariishi et al. 1986). Although this information was available for many of the participants in this study, it was collected > 5 years after the blood drawings. Because these data may not have been representative of the behavior at the time of blood draw, they were omitted from the analysis. Therefore, future studies should collect information on all possible risk factors at the time of blood collection.

High residual error and low explained levels of variation in regression models are common when dealing with human populations because of human variability, and they indicate that there is still unexplained uncertainty in these models. Results of this study demonstrate that spatial dependence in these residuals accounts for some of this error. However, residual spatial variation was recognized in all regression models, suggesting that further investigation of spatial information not considered in this study may improve these models. It is therefore important to collect information not only on potential individual-level risk factors but also on all spatial risk factors when designing future studies. Additional potential risk factors that may have been helpful in this study would have included: BMI, occupation, household and occupational exposure to organochlorines, consumption of local and fatty fish, consumption of homegrown vegetables, recreational swimming in local surface waters, land use, and drinking water well location and/or source aquifer.

Besides accounting for all potential risk factors, future research in this area would benefit from the addition of environmental exposure models. For example, air dispersion or groundwater modeling results could be coupled with biomarkers in assessing the impact of residing near a potential source. These models would take into account wind and groundwater patterns that have the potential to greatly affect contamination at a specific location. Not enough information on the Superfund site studied here was available for such models to be incorporated into our results. This limitation may greatly affect the results of this study because much of the contamination may have been via groundwater and surface water, thereby obscuring the relationship between the site and residence and introducing exposure measurement error.

The study described in this article relies on two assumptions related to participant address information. First, it assumes that participants’ addresses at the time of the blood draw represent their residential location during the time they were most exposed to organochlorines. If there were changes of addresses before blood sampling, and if this exposure measurement error was random, the results may be biased toward the null. It is also possible that they may have had more exposure at their place of employment or recreation than at their residence. Furthermore, we assumed that the locations of the residences were geocoded accurately. However, this assumption is not always valid because there exists positional inaccuracy associated with geocoding using a geographic information system (Bonner et al. 2003; Boscoe et al. 2002; Cayo and Talbot 2003; McElroy et al. 2003). Although this positional inaccuracy was not found to be significant in a study by Bonner et al. (2003), the sensitivity to this bias of the models presented in this article is evaluated elsewhere (Henshaw 2004).

In summary, > 1,200 Superfund sites across the country are contaminated with substances that adversely affect human health (U.S. EPA 2003), and these sites are often located in urban areas surrounded by residences. We presented an analytical approach to investigating the relationship between residential location and possible organochlorine exposure. This approach included spatial information that allowed for the consideration of possible geographic determinants of exposure and regression inference that accounted for possible residual spatial variation.

## Figures and Tables

**Table 1:** Summary of demographic information for study population.

Characteristic	Value
No. of participants	1,374
Age [years (range)]	53.1 ± 0.30 (15–90)
Whites (%)	98.3
Education [years (range)]	11.24 ± 0.08 (1–29)
Current smokers (%)	25.9
Males (%)	57.8
City water drinkers (%)	66.7
Spring water drinkers (%)	0.7
Well water drinkers (%)	11.8
Urban residents (%)	35.8
Distance to site [miles (range)]	3.97 ± 0.12 (0.26–28.33)

Values are percent or mean ± SD.

**Table 2:** Summary of blood organochlorine data for study population.

Organochlorine	No. of samples	Range	Mean ± SD
*p*,*p*-DDE			
Lipid adjusted (ng/g)	1,274	36.4–25,205	3023.5 ± 66.3
Unadjusted (ng/mL)	1,374	0.19–185.9	20.0 ± 0.45
Total of PCB congeners 105, 118, 146, 153, 156, 170, 180			
Lipid adjusted (ng/g)	1,046	129.4–9,355	771.5 ± 22.0
Unadjusted (ng/mL)	1,049	0.73–63.9	5.0 ± 0.15
Dieldrin			
Lipid adjusted (ng/g)	685	1.7–825	113.07 ± 3.05
Unadjusted (ng/mL)	688	0.01–5.6	0.72 ± 0.02

**Table 3:** Results of GLS regression determining the effects of covariates on blood organochlorine levels: parameter coefficients (95% confidence intervals).

	Compounds used at the site				
	DDE			Compounds not used at the site: total PCBs	
Covariate	Lipid unadjusted (ng/mL)	Lipid adjusted (ng/g)	Dieldrin Lipid adjusted (ng/g)	Lipid unadjusted (ng/mL)	Lipid adjusted (ng/g)
Distance from the site to residence (miles)	0.12 (−0.21 to 0.44)	9.1 (−31.8 to 50.0)	−1.6** (−3.1 to −0.1)	NA	NA
Urban vs. rural residence (rural vs. Hagerstown)	NA	NA	NA	0.37 (−0.13 to 0.87)	66.9* (−16.1 to 149.9)
Age (years)	0.12*** (0.05 to 0.20)	10.7* (−0.89 to 22.3)	−0.19 (−0.75 to 0.37)	0.01 (−0.009 to 0.87)	−0.43 (−3.55 to 2.68)
Sex (male vs. female)	10.8*** (9.2 to 12.3)	1,695*** (1,453 to 1,937)	14.6* (−2.5 to 31.7)	4.2*** (3.8 to 4.7)	635*** (567 to 701)
Education (years)	−0.12 (−0.40 to 0.15)	3.8 (−38.7 to 46.3)	−1.8 (−4.2 to 0.6)	−0.06 (−0.13 to 0.02)	−5.3 (−17.4 to 6.8)
Smoking status (nonsmoker vs. smoker)	−2.4** (−4.2 to −0.5)	−296.4** (−580.6 to −12.3)	21.8*** (7.1 to 36.5)	−0.25 (−0.74 to 0.25)	−36.7 (−114.9 to 41.5)
Drinking water source (spring vs. municipal)	15.4*** (6.9 to 23.9)	1918.8*** (600.8 to 3236.8)	221.2*** (152.6 to 289.7)	−0.17 (−2.4 to 2.1)	−83.4 (−437.0 to 270.2)
Drinking water source (well vs. municipal)	2.5* (−0.2 to 5.2)	362.6* (−43.6 to 768.7)	9.4 (−12.1 to 30.9)	0.27 (−0.40 to 0.94)	48.9 (−57.7 to 155.5)
Adjusted *R*^2^	0.209	0.243	0.3376	0.428	0.36

NA, variable not chosen for final model.

* *p*-value < 0.10

** *p*-value < 0.05

*** *p*-value < 0.01.
